# Activation of glucagon-like peptide-1 receptors reduces the acquisition of aggression-like behaviors in male mice

**DOI:** 10.1038/s41398-022-02209-0

**Published:** 2022-10-13

**Authors:** Jesper Vestlund, Qian Zhang, Olesya T. Shevchouk, Daniel Hovey, Lundström Sebastian, Lars Westberg, Elisabet Jerlhag

**Affiliations:** 1 Department of Pharmacology, Institute of Neuroscience and Physiology, The Sahlgrenska Academy at the University of Gothenburg, Gothenburg, Sweden; 2 Institute of Neuroscience and Physiology, Gillberg Neuropsychiatry Centre, University of Gothenburg, Gothenburg, Sweden

**Keywords:** Psychiatric disorders, Molecular neuroscience

## Abstract

Aggression is a complex social behavior, which is provoked in the defense of limited resources including food and mates. Recent advances show that the gut-brain hormone ghrelin modulates aggressive behaviors. As the gut-brain hormone glucagon-like peptide-1 (GLP-1) reduces food intake and sexual behaviors its potential role in aggressive behaviors is likely. Therefore, we investigated a tentative link between GLP-1 and aggressive behaviors by combining preclinical and human genetic-association studies. The influence of acute or repeated injections of a GLP-1 receptor (GLP-1R) agonist, exendin-4 (Ex4), on aggressive behaviors was assessed in male mice exposed to the resident-intruder paradigm. Besides, possible mechanisms participating in the ability of Ex4 to reduce aggressive behaviors were evaluated. Associations of polymorphisms in *GLP-1R* genes and overt aggression in males of the CATSS cohort were assessed. In male mice, repeated, but not acute, Ex4 treatment dose-dependently reduced aggressive behaviors. Neurochemical and western blot studies further revealed that putative serotonergic and noradrenergic signaling in nucleus accumbens, specifically the shell compartment, may participate in the interaction between Ex4 and aggression. As high-fat diet (HFD) impairs the responsiveness to GLP-1 on various behaviors the possibility that HFD blunts the ability of Ex4 to reduce aggressive behaviors was explored. Indeed, the levels of aggression was similar in vehicle and Ex4 treated mice consuming HFD. In humans, there were no associations between polymorphisms of the *GLP-1R* genes and overt aggression. Overall, GLP-1 signaling suppresses acquisition of aggressive behaviors via central neurotransmission and additional studies exploring this link are warranted.

## Introduction

Aggression is an evolutionary conserved innate social behavior, which is essential for the defense of limited resources like food and mates (for review see ref. [1]). While men and women are aggressive at a similar level, several sex-dependent differences exists [2, 3]. Importantly, men are physically whereas girls more indirectly aggressive [4] and men rather than women show provocation-induced anger [5]. Besides, male gender is a high predictor of violent behaviors, and ~90% of all the convicted violent offenders are males [6, 7]. Therefore, the neurobiology of overt aggression is mostly studied in males or in male rodents using the resident intruder test [8]. While laboratory female mice in general display no or mild aggression in this test, lactating or gestational female mice express a robust aggressive behavior [9, 10]. As other neurobiological underpinnings most likely are central for aggression in these mice, males are often used in the resident intruder test [8]. In humans, excessive overt aggression is associated with death, crimes, and drug use [11] as well as contributes to the burden of various psychiatric disorders [12, 13]. Hence, preclinical studies exploring mechanisms central for aggressive behaviors in male mice are thus warranted in attempt to understand this complex disease [11, 14].

Although vast signaling pathways have been identified as central for aggression in male rodents additional underlying pathways remain to be determined. When it comes to brain regions, those central for reward such as the nucleus accumbens (NAc) [15–18], ventral tegmental area (VTA) [19, 20], hippocampus [21], amygdala [22–24], prefrontal cortex [25] appear central also for aggression. Another area processing reward is the paraventricular thalamus (PVT), which also is implicated in aggressive behaviors [26]. Supportively, winning a fight is highly rewarding in male rodents [27]. In contrast to males, female mice display a defensive/reactive rather than rewarding phenotype at aggression [28]. As aggression is a multifaceted behavior, other brain regions and phenotypes are involved. Indeed, the initiation of aggression engages brain areas like hypothalamus, specifically bed nucleus of the stria terminalis (BNST) and medial preoptic area (MPOA) [29–31]. Dorsal raphe, the main area responsible for serotonin production, is yet another area crucial for initiating aggressive behaviors (for reviews see ref. [1, 32]). Indeed, both rodent and human studies reveal that serotonin modulate aggressive behaviors. The monoamines noradrenalin and dopamine are other neurotransmitters mediating aggressive behaviors. Although hormones such as testosterone and corticosterone modulate aggression (for reviews see ref. [1, 32]), another hormone of importance is the gut-brain peptide ghrelin, which enhances such behaviors in male mice [33, 34].

Another gut-brain hormone is glucagon-like peptide-1 (GLP-1), which is well-known for its ability to suppress food intake, appetite, or body weight and to modulate glucose homeostasis (for review see [35]). These physiological properties have contributed towards the approval of GLP-1 receptor (GLP-1R) agonists for treatment of diabetes type 2 [36] and obesity [37]. Besides metabolic homeostasis, GLP-1R agonists like exendin-4 (Ex4) attenuate reward from addictive drugs and palatable food in male rodents [38–42]. More recent studies reveal that GLP-1R regulates social behaviors as Ex4 decrease sexual behaviors in male mice [43, 44]. Aggression is another social behavior driven by the defense of food and mates, consummatory behaviors regulated by GLP-1 signaling (for review see refs. [1, 45]). Together with the findings that obesity and aggression share overlapping genes [46] and are co-morbid in epidemiological studies [47, 48], indicate that obesity and aggression share common risk factors; plausibly GLP-1 signaling. Indeed, the potential role of GLP-1R for aggression is unknown.

To explore this knowledge gap, we herein investigated the link between GLP-1R activation and aggressive behaviors by combining preclinical experiments and a human genetic-association study. We investigated whether acute or repeated treatment with Ex4 decreases the expression and acquisition of aggressive behavior in male mice in the resident-intruder paradigm. Peripheral and central mechanisms contributing towards the ability of Ex4 to reduce aggressive behaviors were then evaluated by means of various molecular biology and biochemical methods. First, the ex vivo levels of monoamines in aggression-related brain areas and the plasma concentrations of corticosterone and testosterone were measured in male mice treated with Ex4 or vehicle during the resident intruder test. Besides, the levels of noradrenalin, and serotonin-related proteins and the counts of FOS immunoreactivity, a marker of neuronal activity, was determined.

Exposure to high-fat diets (HFD) reduces preference for palatable foods, depression-like behaviors and drug reward, a decline plausibly involving altered dopaminergic neurotransmission [49–51]. Besides, in male mice HFD enhances aggression [52] and impairs the responsiveness to GLP-1 on various behaviors [53–55]. Additional pilot resident intruder experiments therefore assessed the possibility that HFD (*i.e*., peanut butter) blunts the ability of Ex4 to reduce aggressive behaviors. Additionally, the protein levels of noradrenalin, and serotonin-related proteins of these mice were assessed. Finally, we assessed the association between polymorphisms in *GLP-1R* genes and self-reported overt aggression in young men from the Child and Adolescent Twin Study in Sweden (CATSS).

## Material and methods

Herein a combination of preclinical experiments and a human genetic study are used to study the hypothesis that activation of GLP-1R suppresses aggression behaviors. Indeed, the resident-intruder model is a valid model to study aggressive behaviors directed towards others (overt aggression) in male mice [8]. As genetic vulnerability contributes to the expression of overt aggression in humans (for review see ref. [56]), the identification of genes associated with this behavior can be useful when studying mechanisms underlying aggression.

### Preclinical study

#### Animals

To establish a robust aggressive behavior in the resident mouse (C57Bl/6 N), an intruder mouse of a smaller, submissive strain 129/SvEv was used [33]. The animals and their situation were identical to our previous study [33]. The conducted experiments were approved by the Swedish Ethical Committee on Animal Research in Gothenburg (Ethical number: 3348/20; 96/15). Males were studied as they show a robust response in the resident intruder test, whereas laboratory female mice in general display no or low aggression in this test and as lactating or gestational female mice with higher aggression most likely involve other neurobiological substrates [8–10, 14].

#### Drugs and peanut butter

Two different doses of Ex4 (1.2 µg/kg or 2.4 µg/kg; Tocris Bioscience, Abingdon, United Kingdom) dissolved in vehicle (0.9% NaCl) were injected intraperitoneal (IP) 10 min prior to the test [39]. The selected doses were used as they do not alter locomotor behavior in an open field test per se, whereas the higher one blocks the alcohol-induced locomotor stimulation in male mice [39]. Peanut butter (Green Choice; Coop, Gothenburg, Sweden) was used.

#### Resident intruder paradigm

The resident intruder paradigm was used in order to evaluate the effect of acute or repeated administration of Ex4 on aggressive behaviors, and to explore the possible influence of peanut butter on such behavioral outcome. The resident intruder paradigm is a valid model of overt aggression, where a male mouse defends his territory against a conspecific intruder [8, 14]. It was conducted as described in previous studies [8, 33, 57, 58]. Importantly, all mice (resident and intruder) had full access to regular chow throughout the experimental setup, except during the daily resident intruder encounter (maximum 10 min per day). The mice were not food restricted as we aim to explore the effect of Ex4 during regular physiological conditions. Besides, after food restriction access to food during the resident intruder test decrease aggressive behaviors [59], a possible confounding factor eliminated in the present design.

#### Experimental designs

The designs of the test differ as they either investigate the effect of acute or repeated Ex4 treatment on aggressive behavior.

**Fig. 1:**
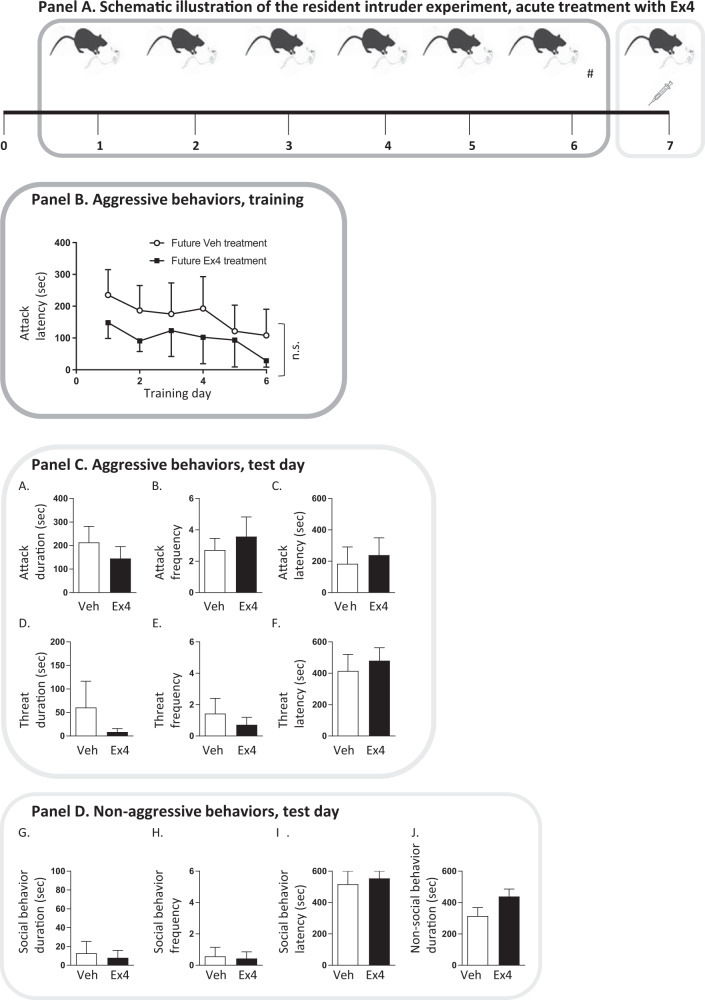
Acute exendin-4 injection did not alter aggressive behaviors in male mice. Panel A illustrates the outline of the resident intruder experiment with an acute exendin-4 (Ex4) treatment. The mice were trained in the resident intruder paradigm at day 1–6, and were then stratified (#) to receive an acute Ex4 (2.4 µg/kg, IP, *N* = 7) or vehicle (Veh, IP, *n* = 7) injection at test day (day 7). During training the latency to attack was recorded, allowing similar attack score during training between future treatment groups (Panel B). At the test day, aggressive behaviors (attack, threat; Panel C) and non-aggressive behaviors (social and non-social; Panel D) were scored for 10 min. As shown in Panel B, there are no differences in attack latency during training between mice assigned into the two treatment groups at test day (treatment F(1,12) = 0.52, *P* = 0.4856, time F(5,60) = 3.67, *P* = 0.0058, interaction F(5,60) = 0.37, *P* = 0.8691). As evident in Panel C, there are no differences between mice treated acutely with Ex4 or vehicle when it comes to (**A**) attack duration (t(12) = 0.81, *P* = 0.4359), (**B**) attack frequency (t(12) = 0.59, *P* = 0.5672), (**C**) attack latency (t(12) = 0.36, *P* = 0.7228), (**D**) threat duration (t(12) = 0.92, *P* = 0.3750), (**E**) threat frequency (t(12) = 0.66, *P* = 0.5215) or (**F**) threat latency (t(12) = 0.49, *P* = 0.6330). In comparison to vehicle Ex4 did not alter non-aggressive behaviors (Panel D), as Ex4 did not alter the (**G**) duration (t(12) = 0.32, *P* = 0.7553), (**H**) frequency (t(12) = 0.20, *P* = 0.8448), (**I**) latency (t(12) = 0.39, *P* = 0.7057) of social behaviors or (**J**) the duration of non-social behaviors (t(12) = 1.75, *P* = 0.1059). Data are presented as mean ± SEM.

Experiment 1 (Fig. 1, Panel A) was designed to study the effect of an acute injection of Ex4 on aggressive behavior in mice with an established aggression. Therefore, only mice displaying aggressive behavior on day 6 was included in the statistical analysis. Therefore, two non-aggressive mice were excluded from the analysis. After six initial training days the resident mice were divided into two future treatment groups, where the mice had similar attack latency during day 1–6. Thereafter, the mice received either an acute Ex4 (2.4 µg/kg, IP) or vehicle injection on the final test day (day 7).

In contrast to experiment 1, low aggression was not an exclusion criterion for experiment 2–5 as these evaluate the possibility that repeated Ex4 treatment during aggression acquisition dose-dependently reduces aggressive behaviors in male mice. As lower innate level of aggressive-like behavior might confound the obtained data, vehicle controls are included and treatment were either randomized (experiment 2), stratified based on acquired aggression level (experiment 1) or innate aggression level (experiment 3,4,5). In experiment 2 (Supplementary Fig. 1, Panel A), a pilot test, the resident mice were randomized to future treatment prior to training (day 0). The resident mice were then injected with either Ex4 (2.4 µg/kg, IP) or vehicle on each training (day 1–6) and on the test day (day 7).

**Fig. 2:**
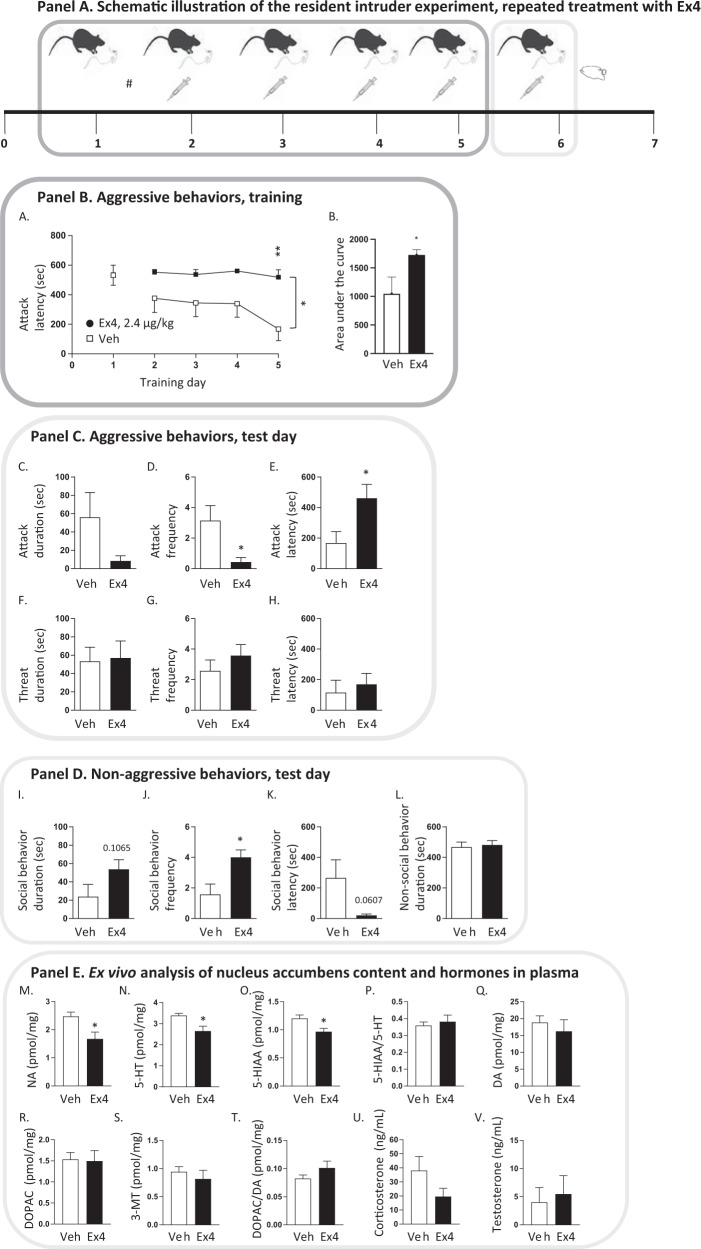
Repeated exendin-4 treatment suppressed the acquisition of aggressive behaviors in male mice. A schematic illustration of the resident intruder experiment (Panel A) with repeated exendin-4 (Ex4) treatment. At day 1, the mice were trained in the resident intruder paradigm and the time to first attack was scored. This allows stratification (#; with similar attack score between future treatment groups) into Ex4 (2.4 µg/kg, IP, *N* = 7) or vehicle (Veh, IP, *n* = 7) injections throughout training (day 2–5) and at the test day (day 6). During the training, the latency to attack was recorded (Panel B), and aggressive behaviors (attack, threat, Panel C) and non-aggressive behaviors (social and non-social, Panel D) were scored for 10 min at the test day. Directly after the test the mice were euthanized, and the brains and plasma from each mouse were collected for ex-vivo analyses (Panel E). **A** In these mice the baseline latency to attack was similar between future treatment groups (t(12) = 0.04, *P* = 0.9687). **A** Repeated treatment with Ex4 during training increased the latency to attack (treatment F(1,12) = 8.10, *P* = 0.0148, time F(3,36) = 4.09, *P* = 0.0134, interaction F(3,36) = 2.11, *P* = 0.1163; *n* = 7 per group). Post-hoc analysis reveal that this difference is evident at day 5 (*P* = 0.0023). **B** This is further evident as the area under the curve for attack latency during training is higher in Ex4 treated mice compared to those treated with vehicle (t(12) = 2.20, *P* = 0.0455). At test day, Ex4 reduced aggressive behaviors (Panel C), and enhanced social behaviors (Panel D). Compared to vehicle, Ex4 (**C**) did not affect the attack duration (t(12) = 1.71, *P* = 0.1131), but (**D**) reduced attack frequency (t(12) = 2.64, *P* = 0.0218) and (**E**) increased attack latency (t(12) = 2.49, *P* = 0.0286). Ex4 treated mice had no alterations in threat (**F**) duration (t(12) = 0.15, *P* = 0.8824), (**G**) frequency (t(12) = 0.98, *P* = 0.3448), (**H**) latency, (t(12) = 0.50, *P* = 0.6291) compared to vehicle-treated mice. Ex4 did not alter (**I**) social behavior duration (t(12) = 1.75, *P* = 0.1065), but (**J**) increased social behavior frequency (t(12) = 2.89, *P* = 0.0136) and (**K**) tended to reduce the social behavior latency (t(12) = 2.07, *P* = 0.0607). Ex4 did not affect (**L**) non-social behavior duration (t(12) = 0.33, *P* = 0.7482). In an attempt to define underlying mechanisms, the levels of monoaminergic neurotransmission in various aggression-related areas and the plasma levels of testosterone or corticosterone were investigated in these mice (Panel E). Compared to vehicle, repeated Ex4 injections to mice in the resident intruder test decreased (**M**) noradrenalin (NA) (t(12) = 2.78, *P* = 0.0168), (**N**) serotonin (5-HT) (t(12) = 2.91, *P* = 0.0132) and (**O**) 5-HIAA (t(12) = 2.83, *P* = 0.0152) levels in nucleus accumbens (NAc). Contrarily, there were no differences between treatments when it comes to (**P**) 5-HT turnover (t(12) = 0.53, *P* = 0.6027), (**Q**) dopamine (DA) (t(12) = 0.66, *P* = 0.5236), (**R**) DOPAC (t(12) = 0.14, *P* = 0.8898), (**S**) 3-MT (t(12) = 0.72, *P* = 0.4849) levels or (**T**) DA turnover (t(12) = 1.36, *P* = 0.1977) in NAc.In these mice, Ex4 (*n* = 6, one sample excluded due to contamination) does not alter the levels of (**U**) corticosterone or (**V**) testosterone in plasma compared to vehicle (*n* = 7). The plasma concentrations of (**U**) corticosterone (t(11) = 1.52, *P* = 0.1571) and (**V**) testosterone (t(11) = 0.35, *P* = 0.7300) did not differ between mice treated with Ex4 (*n* = 6, one sample contaminated following handling) or vehicle (n = 7) from these experiments. Data are presented as mean ± SEM; significant data are illustrated by *P < 0.05, **P < 0.01.

In experiment 3 (Fig. 2, Panel A), the resident mice were untreated during the first training day (day 1), and were thereafter stratified to future treatment groups with similar baseline attack latency. The resident mice were injected with either Ex4 (2.4 µg/kg, IP) or vehicle on each training (day 2–5) and on the test day (day 6). Thereafter the mice were euthanatized and brains (measurements of monoamine levels in aggression-related areas) as well as plasma (determination of corticosterone and testosterone concentrations) were collected. In this experiment, two mice were excluded due to repeated jumping out of the cage or to violent behavior already at day 1.

**Fig. 3:**
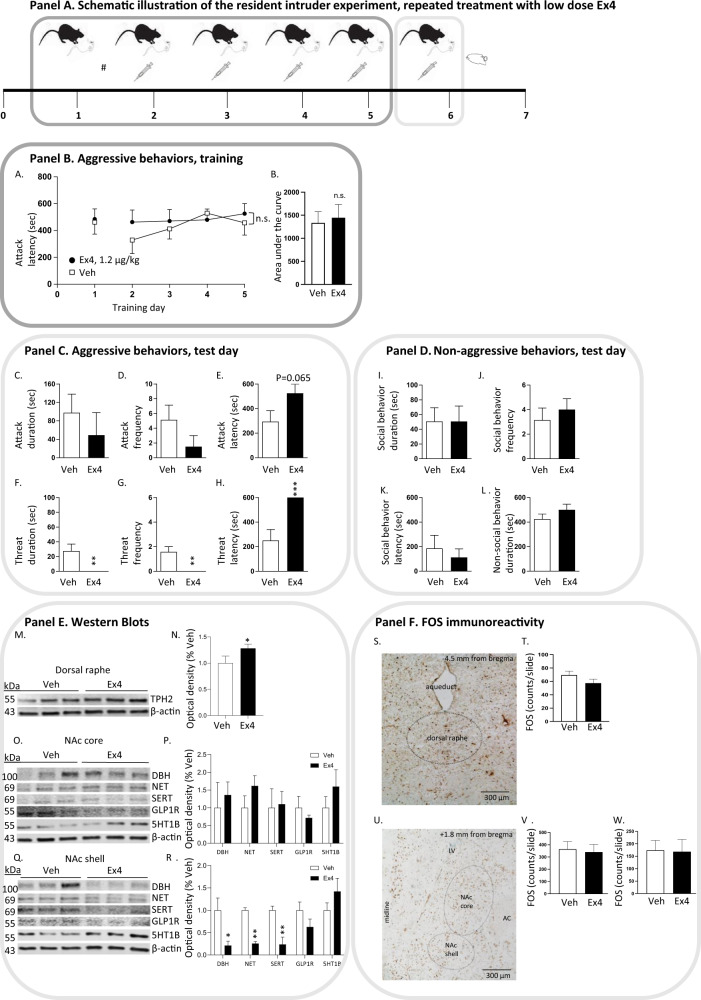
Repeated treatment with a low dose of exendin-4 reduces aggressive behaviors in male mice. The outline of the resident intruder experiment with a low dose of exendin-4 (Ex4) is illustrated in Panel A. At day 1, the mice were trained in the resident intruder paradigm and the time to first attack was scored. This allows stratification (#; with similar attack score between future treatment groups) into Ex4 (1.2 µg/kg, IP, *n* = 8) or vehicle (Veh, IP, *n* = 7) injections throughout training (day 2–5) and at the test day (day 6). During training the latency to attack was recorded (Panel B), and aggressive behaviors (attack, threat, Panel C) and non-aggressive behaviors (social and non-social, Panel D) were scored for 10 min at the test day. Directly after the test, the mice were euthanized, and the brains from each mouse were collected for ex-vivo analyses (Panel E, F). **A** In these mice the baseline latency to attack was similar between future treatment groups (t(13) = 0.20, *P* = 0.8476). **A** A low dose of Ex4 did not alter the latency to attack (treatment F(1,13) = 0.31, *P* = 0.5850, time F(3,39) = 1.64, *P* = 0.1959, interaction F(3,39) = 0.96, *P* = 0.4221; *n* = 8, Ex4, *n* = 7, veh), possibly implying that higher doses of Ex4 are required to influence attack latency during the training. **B** This is further evident as the area under the curve was similar between vehicle and Ex4 treated mice (t(13) = 0.30, *P* = 0.7704). At test day, a low dose of Ex4 affected some aggressive (Panel C), but not any non-aggressive (Panel D) behaviors. Ex4 (**C**) did not alter attack duration (t(13) = 0.75, *P* = 0.4646), or (**D**) attack frequency (t(13) = 1.48, *P* = 0.1619), but (**E**) tended to enhance the or attack latency (t(13) = 2.02, *P* = 0.0650). In addition, Ex4 (**F**) reduced threat duration (t(13) = 3.15, *P* = 0.0077), (**G**) suppressed threat frequency (t(13) = 3.94, *P* = 0.0017) and (**H**) enhanced threat latency (t(13) = 4.24, *P* = 0.0010). The low dose of Ex4 did not alter (**I**) social behavior duration (t(13) = 0.002, *P* = 0.9982), (**J**) social behavior frequency (t(13) = 0.65, *P* = 0.5281), (**K**) social behavior latency (t(13) = 0.59, *P* = 0.5639) or (**L**) non-social behavior duration (t(13) = 1.21, *P* = 0.2471). Western blot (Panel E) show (**M**) the expression of tryptophan hydroxylase (TPH2) of raphe from mice of the above resident intruder test, (**N**) and the TPH2 levels are higher after a low dose of Ex4 (*n* = 3) compared to vehicle (*n* = 3). Western blots show the expression of dopamine-β-hydroxylase (DBH), noradrenalin reuptake transporter (NET), serotonin reuptake transporter (SERT), glucagon-like peptide-1 receptor (GLP1R), serotonin-1B-receptor (5HT1B) of (**O**) nucleus accumbens (NAc) core, (**P**) where no differences are evident between treatments, or (**Q**) NAc shell. **R** Compared to vehicle, a low dose of Ex4 reduces the protein levels of DBH, NET, SERT, without alerting the levels of GLP1R, or 5HT1B in NAc shell. β-actin was always used as a loading control. The relative density of the target protein band was normalized to the density of the β-actin band to represent the relative expression of the target protein. FOS immunoreactivity (Panel F) of one representative picture of (**S**) dorsal raphe and (**U**) NAc core and shell, where the FOS positive cells were quantified. Compared to vehicle (*n* = 4, and 2–4 slices from each animal) the low dose of Ex4 (*n* = 3, and 2–4 slices from each animal) did not alter the number of cells positive for FOS immunoreactivity in (**T**) dorsal raphe (**V**) NAc core (**W**) NAc shell. Lateral ventricle (LV), anterior commissure (AC). Data are presented as mean ± SEM; significant data are illustrated by *P < 0.05, **P < 0.01, ***P < 0.001.

Experiment 4 (Fig. 3) was conducted to explore if a low dose of Ex4, as the high dose, reduces aggressive behaviors. Therefore, the resident mice were untreated during the first training day (day 1), and were thereafter stratified to future treatment groups with similar baseline attack latency. The resident mice were injected with either Ex4 (1.2 µg/kg, IP) or vehicle on each training (day 2–5) and on the test day (day 6). After the test, the mice were euthanatized and brains (determination of noradrenalin/serotonin-related proteins or FOS immunoreactivity) were collected. One mouse was excluded as its body weight was lower than the body weight of the submissive mice.

**Fig. 4:**
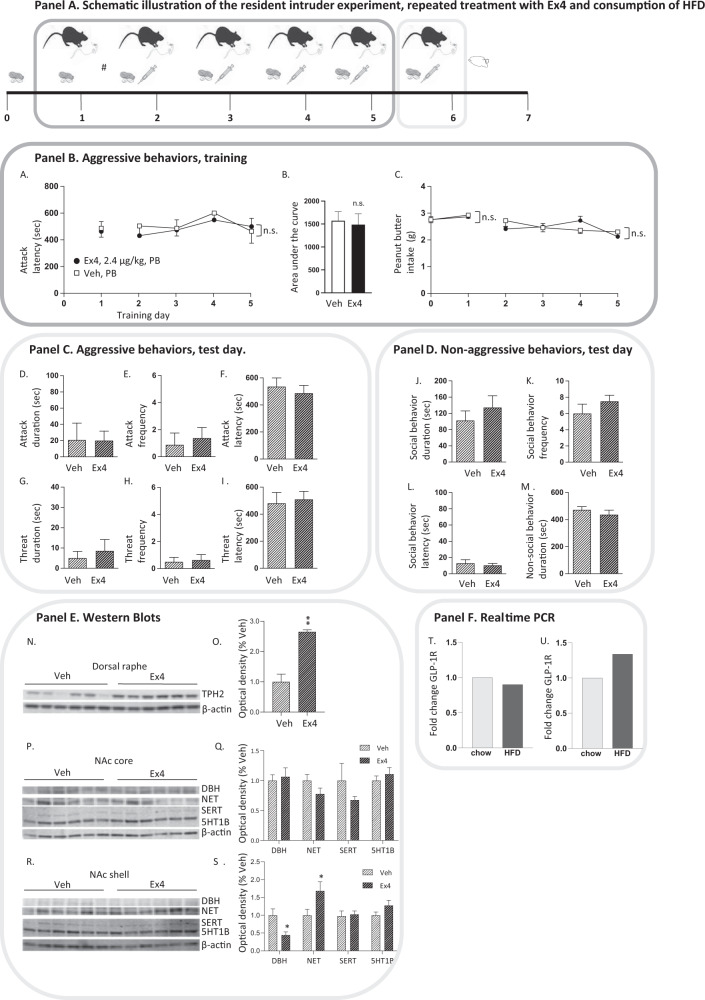
Peanut butter consumption blunts the ability of exendin-4 to decrease aggressive behaviors in male mice. Panel A Illustrates the outline of the resident intruder experiment when mice were fed peanut butter (, day 0–6) and treated repeatedly with exendin-4 (Ex4; 2.4 µg/kg, IP, *n* = 8) or vehicle (Veh, IP, *n* = 8). At day 1, the mice were trained in the resident intruder paradigm and the time to first attack was scored. This allows stratification (*; with similar attack score between future treatment groups) into Ex4 injections throughout the rest of training (day 2–5) and at the test day (day 6). During training the latency to attack was recorded (Panel B), and at the test day aggressive behaviors (attack, threat, Panel C) and non-aggressive behaviors (social and non-social, Panel D) were scored for 10 min. Directly after the test the mice were euthanized, and the brains from each mouse were collected for ex-vivo analyses (Panel F). **A** In these mice the baseline latency to attack was similar between future treatment groups (t(14) = 0.23, *P* = 0.8186). As described above, mice fed with chow display less aggressive behaviors after repeated administration of Ex4 (Fig. 2 and Fig. 3). In contrast, mice fed with HFD (i.e., peanut butter) neither exhibit altered latency to attack during training (Panel B), nor reduced aggressive behaviors at test (Panel C) after repeated administration of Ex4 (2.4 µg/kg, IP). **A** Specifically, the latency to attack was similar in HFD fed mice treated with either vehicle of Ex4 (treatment F(1,14) = 0.1470, *P* = 0.7072, time F(3,42) = 1.66, *P* = 0.1892, interaction F(3,42) = 0.3804, *P* = 0.7676; *n* = 8 per group). **B** Besides, the area under curve of attack latency is similar between the two groups of mice (t(14) = 0.27, *P* = 0.7931). **C** Moreover, the 24-h peanut butter intake was similar in vehicle and Ex4 treated mice prior to (treatment F(1,14) = 0.05, *P* = 0.8343, time F(1,14) = 1.83, *P* = 0.1981, interaction F(1,14) = 0.10, *P* = 0.7517) and during (treatment F(1,14) = 0.02, *P* = 0.8789, time F(3,42) = 3.36, *P* = 0.0275, interaction F(3,42) = 2.78, *P* = 0.0530) training. At test day, there were no differences between treatments when it comes to (**D**) attack duration (t(14) = 0.03, *P* = 0.9758), (**E**) attack frequency (t(14) = 0.43, *P* = 0.6758), (**F**) attack latency (t(14) = 0.56, *P* = 0.5863), (**G**) threat duration (t(14) = 0.54, *P* = 0.5984), (**H**) threat frequency (t(14) = 0.87, *P* = 0.8178) or (**I**) threat latency (t(14) = 0.29, *P* = 0.7769). Neither was there a difference in (**J**) social behavior duration (t(14) = 0.86, *P* = 0.4020), (**K**) social behavior frequency (t(14) = 1.09, *P* = 0.2940), (**L**) social behavior latency (t(14) = 0.47, *P* = 0.6467) or (**M**) social behavior duration (t(14) = 0.87, *P* = 0.4003) between vehicle and Ex4 treated mice. Western blot (Panel E) show (**N**) the protein levels of tryptophan hydroxylase (TPH2) of raphe from mice of the above resident intruder test, (**O**) and the TPH2 levels are higher after a low dose of Ex4 (*n* = 6) compared to vehicle (*n* = 6) in mice fed peanut butter. Western blots show the protein levels of dopamine-β-hydroxylase (DBH), noradrenalin reuptake transporter (NET), serotonin reuptake transporter (SERT), serotonin-1B-receptor (5HT1B) of (**P**) nucleus accumbens (NAc) core, (**Q**) where no differences are evident between treatments, or (**R**) NAc shell. (**S)** Compared to vehicle, Ex4 reduces the protein levels of DBH, and increases NET, without altering the protein levels of GLP1R, or 5HT1B in NAc shell. β-actin was always used as a loading control. The relative density of the target protein band was normalized to the density of the β-actin band to represent the relative expression of the target protein. The real time PCR data (Panel F) reveal that the expression of the *GLP-1R* in (**T**) NAc (t(10) = 0.33, *P* = 0.7437) or (**U**) dorsal raphe (t(10) = 1.22, *P* = 0.2516) was similar in mice exposed to chow compared to those exposed to HFD. Data are presented as mean ± SEM; significant data are illustrated by *P < 0.05, **P < 0.01.

As HFD impairs the responsiveness to GLP-1 on various behaviors the possibility that HFD blunts the ability of Ex4 to reduce aggressive behaviors was explored in experiment 5 (Fig. 4). The resident mice had free access to HFD (i.e., peanut butter) and chow in their home cage prior to the resident intruder test, allowing acclimatization to HFD. The mice were untreated during the first training day (day 1), and were thereafter stratified to future treatment groups with similar baseline attack latency. The resident mice were injected with Ex4 (2.4 µg/kg, IP) or vehicle on each training (day 2–5) and on the test day (day 6). Each day, the pre-weighed peanut butter was introduced to the mice after resident intruder test removed and weighed prior to the following test (24-h intake). The mice had access to HFD, but not chow, during the daily encounter (maximum 10 min per day). Neither the dominant or submissive mice appeared to eat HFD during the resident intruder test as evident by visual observation. In fact, access to food during test in combination with food restriction decrease aggression behavior in the resident intruder test [59], the visual inspection in combination with free access to food indicate that the mice were not hungry when performing the test. After the test day, the mice were euthanatized and brains (measurement of serotonin/noradrenalin-related proteins) were collected.

#### Ex-vivo biochemical analyses

After experiment termination, tissues were collected and placed on dry ice and frozen at −80 °C until analysis as described before [33]. Ex vivo measurements (HPLC-EC, ELISA, western blot, or immunohistochemistry) were conducted as they allow determination of downstream mechanisms responsible for the ability of Ex4 to reduce aggressive behavior. Besides, the effect of HFD exposure on the expression of *GLP-1R* was examined by means of reverse transcription and real-time PCR.

An established HPLC-EC system [33, 60] was used to measure monoamines in important brain areas (Table 1) from mice treated with Ex4 (2.4 µg/kg). The plasma levels of corticosterone or testosterone, important for aggression (for reviews see refs. [1, 32]), was measured in these mice with a specific Enzo ELISA kit (AH diagnostics, Stockholm, Sweden) as described previously [33].

**Table 1:** Selected important areas for aggressive behaviors.

Brain region	Rational for selection	Reference
Hypothalamus (sub-regions BNST, MPOA)	Initiation of aggressive behaviors	for review see ref. [1]
Nucleus accumbens, ventral tegmental area, prefrontal cortex, hippocampus and amygdala	Reward of aggressive behaviors	for review see ref. [1]
Paraventricular thalamus	Reward of aggression	[2]

Description of the role of the selected brain areas central for aggression, with key references. Bed nucleus of the stria terminalis (BNST) and medial preoptic area (MPOA).

Two separate western blot studies were conducted from brains collected from mice of the resident intruder test i) treated with Ex4 (1.2 µg/kg) or vehicle and eating chow or ii) treated with Ex4 (2.4 µg/kg) or vehicle and exposed to HFD during test. Western blot of serotonin or noradrenalin proteins in NAc, specifically the core and shell compartments, were conducted as the neurochemical data reveal that these mediate the Ex4 reduced aggression. Specifically, for NAc shell both the ventral and medial subregions were selected. Moreover, dorsal raphe was explored as this area provides the main serotoninergic projections to NAc. Detailed protocol described in Table 2. After being blocked with 5% skim milk for 1 h at room temperature, the membrane was sequentially incubated with primary/secondary antibodies according to Table 3. β-actin was used as a loading control. The relative density of the target protein band was normalized to the density of the β-actin band to represent the relative expression of the target protein.

**Table 2:** Methods for western blot.

Procedure	Protocol
Extraction of proteins from punched tissues	RIPA lysis buffer (25 mM Tris (pH 7.4), 150 mM NaCl, 1% Triton X-100, 1% sodium deoxycholate, and 0.1% SDS) mixed with protease inhibitor cocktail (one tablet in 10 ml, Roche Diagnostics GmbH, Mannheim, Germany).
Evaluation of the total protein concentration	Bicinchoninic acid (BCA) protein assay (#23228, Thermo Scientific, Rockford, IL) following the manufacturer’s instructions
Separation of an equal amount (30 µg per lane) of protein loaded on each well	SDS-PAGE followed by the electrophoretic transfer of protein bands onto a polyvinylidene difluoride (PVDF) membrane

Detailed description of the Western Blot procedure and protocol used in the present study.

**Table 3:** Selected proteins, antibodies, dilutions.

Target protein	Primary antibody (COMPANY, City, Country)	Dilution/incubation	Secondary antibody (COMPANY, City, Country)	Dilution/incubation
TPH2	Rabbit anti-TPH2 LS‑C346253, Lifespan Bio, Seattle, WA, USA	1:500 in 5% skim milk/ overnight	Scanlater Eu-labeled Goat Anti- Rabbit lgG R8029 Molecular Devices, Sunnyvale, CA, USA	1:5000 in 5% skim milk/ RT 1 h
DBH	Rabbit anti-DBH b209487, Abcam, Cambridge, UK	1:500 in 5% skim milk/ overnight	Scanlater Eu-labeled Goat Anti-Rabbit lgG R8029 Molecular Devices, Sunnyvale, CA, USA	1:5000 in 5% skim milk/ RT 1 h
NET	Rabbit anti-NET LS-C408311, Lifespan Bio, Seattle, WA, USA	1:1000 in 5% skim milk/ overnight	Scanlater Eu-labeled Goat Anti- Rabbit lgG R8029 Molecular Devices, Sunnyvale, CA, USA	1:5000 in 5% skim milk/ RT 1 h
SERT	Rabbit anti-SERT ab9726, Sigma Aldrich, Saint Louis, MO, USA	1:500 in 5% skim milk/ overnight	Scanlater Eu-labeled Goat Anti-Rabbit lgG R8029 Molecular Devices, Sunnyvale, CA, USA	1:5000 in 5% skim milk/ RT 1 h
5HT1B	Rabbit anti-5HT1B SAB4501471, Sigma Aldrich, Saint Louis, MO, USA	1:500 in 5% skim milk/ overnight	Scanlater Eu-labeled Goat Anti-Rabbit lgG R8029 Molecular Devices, Sunnyvale, CA, USA	1:5000 in 5% skim milk/ RT 1 h
GLP1R	Rabbit anti-GLP1R PA5–70645, Invitrogen, Waltham, MA, USA	1:500 in 5% skim milk/ overnight	Scanlater Eu-labeled Goat Anti-Rabbit lgG R8029 Molecular Devices, Sunnyvale, CA, USA	1:5000 in 5% skim milk/ RT 1 h
β-actin	Mouse anti-β-actin #3700, Cell Signaling, Danvers, MA, USA	1:1000 in 5% skim milk/ overnight	Scanlater Eu-labeled Goat Anti-Mouse lgG R8028 Molecular Devices, Sunnyvale, CA, USA	1:5000 in 5% skim milk/ RT 1 h

Description of the selected proteins as well as primary/secondary antibody, dilutions and incubation times used. Tyrosine Hydroxylase Isoforms 2 (TPH2), Dopamineβ Hydroxylase (DBH), Norepinephrine Transporter (NET), Serotonin Transporter (SERT), 5-hydroxytryptamine receptor 1B (5HT1B), Glucagon-like peptide 1 receptor (GLP1R).

The number of FOS positive cells were counted in brain slices from mice treated with Ex4 (1.2 µg/kg; *n* = 3) or vehicle (*n* = 3–4) from the resident intruder experiment (Fig. 3A) as described in detail elsewhere [61]. NAc, specifically the core and shell regions, was assessed as neurotransmission and protein content in this area was associated with aggressive behaviors. As for the Western Blot studies, the ventral rather than medial subregion was selected. To highlight the possibility that Ex4 alters neuronal activation in dorsal raphe responsible for the main serotonin to NAc, the number of FOS positive cells was counted in this area. Besides, additional areas important for aggression was analyzed (Table 2). A series of 2 pictures per brain region of each section was obtained by a Nikon light microscope (Nikon eclipse 90i, Tokyo, Japan), captured with a Nikon DS-Fi1 camera (Nikon, Tokyo, Japan) (Table 4). Cell bodies positive for FOS immunoreactivity (dark brown) of each selected brain region were quantified using Image J-FIJI (National Institutes of Health, Bethesda, MD). The cells were counted automatically using a macro that subtracted background intensity, extracted DAB staining using color deconvolution, set the intensity threshold (same for all pictures from both vehicle and treatment group) and analyzed particles with the size 20–250 pixels squared and with a circularity of 0.7–1.0.

**Table 4:** Coordinate(s) for the selected brain regions sliced for the immunohistochemical staining.

Brain region	Coordinate(s)
Nucleus accumbens	+1.8 and +0.9
Paraventricular thalamus	+0.2
Bed nucleus of the stria terminalis	+0.2
Medial preoptic area	+0.2 and −0.2

Coordinates are presented in mm from bregma.

The expression of *GLP-1R* was explored in NAc and dorsal raphe after 7 days of access to either chow (*n* = 18) or the combination of HFD and chow (*n* = 18) in the home cage to male mice. After termination, the NAc and dorsal raphe was collected by punching. Due to optimizing the detection of *GLP-1R*, regions from three mice were pool to one sample (*n* = 6, per group). TRIzol reagent (15596026, Invitrogen ThermoFisher, Uppsala, Sweden) extracted total RNA, which then was purified and homogenized following manufacturer’s instructions. After adding chloroform, the samples were mixed, incubated (3 min, room temperature) and centrifuged (15 min, 12,000 g, 4 °C). Isopropanol was added to the RNA containing aqueous phase, then incubated (10 min, room temperature) and centrifuged (10 min, 12,000 g, 4 °C). After removal of the supernatant, ethanol (75%) washed the RNA pellet and this was centrifuged (5 min, 7500 g, 4 °C). After removing the supernatant, the RNA was resuspended in DEPC-treated water. RNA (2 μg) were reverse transcribed in Quantiscript reverse transcriptase (20 μl, 205310, QIAGEN) that was incubated (15 min at 42 °C; 3 min at 95 °C). 20 μl samples at triplicates in a TaqMan Fast Advanced Master Mix kit on an ABI QuantStudio 7 Pro Real-Time PCR System was used. cDNA (2 μl) was mixed with TaqMan Gene Expression Assay (1 μl), TaqMan Fast Advanced Master Mix (10 l, 4444553, ThermoFisher). The PCR program was initiated at 95 °C for 2 min, followed by 40 thermal cycles of 1 s at 95 °C and 20 s at 60 °C. A melting curve for primer validation and a template standard curve were performed to show template independent amplification results. The comparative CT method (ABI technical manual) was used to analyze the real-time PCR. The expression of GLP1R(Mm00445292, ThermoFisher) were normalized to the geometric mean of β-actin (Mm01205647, ThermoFisher) and expressed as fold change (ΔΔCt) relative to chow.

#### Statistical analysis

The effects of Ex4 on behaviors and biochemistry data were analyzed by a two-tailed unpaired *t*-test. Moreover, the two-tailed parametric Pearson correlation test was used to assess the correlation between biochemistry data and behaviors. The effect of Ex4 treatment on acquisition of aggression behavior and peanut butter consumption were analyzed using a repeated measure two-way ANOVA followed by a Bonferroni post-hoc test. The bands from western blotting were quantified and analyzed by Image J (Version 1.47; Research Services Branch, National Institute of Mental Health, Bethesda, MD, USA). Comparison between two groups was performed using unpaired *t*-test. Also, the FOS immunohistochemistry and real-time PCR (ΔCt) data were analyzed with an unpaired *t*-test. All data are presented as mean ± SEM. A GraphPad Prism version 7.03 (GraphPad Software, San Diego, CA, USA) was used for all preclinical statistical analyses. A probability value of *P* < 0.05 was considered as statistically significant. Data were analyzed by individual blinded to treatment.

### Human genetic study

#### Participants

The included individuals at age of 18, scored for on-aggressive antisocial and aggressive behavior using the self-reported delinquency questionnaire (response rate = 50%), are from a subsample from CATSS, an on-going study of Swedish twins [62, 63]. A panel of 47 SNPs defined zygosity [64]. Men (*n* = 788) were included in the human genetic study as men, compared to women, display a substantially higher degree of provocation-induced [5] physical aggression [4]. Besides, overt-aggression is less frequent in women of CATSS and the lower variation creates a reduced power of this sub-scale [63]. After exclusion of four individuals (two with documented brain damage, and two non-Caucasian) a total of 784 men were included in the present study. The Ethics Committee at Karolinska Institute approved the study, and all participants signed an informed consent or consented by participating in the interview (Ethical number: 2008-849-31; 2010-1410-31-1; 2016–2135; 2020–04464).

#### Self-reported delinquency questionnaire

The modified self-reported delinquency questionnaire (SRD) was used [65, 66], as described extensively elsewhere [33]. As before [63, 67], the subscale of overt aggression was selected as outcome measure as it better reflects the preclinical findings (Supplementary Table 1). In an attempt to justify the results, extreme outliers were winsorized (mean ± 2 standard deviations; Cronbach’s alpha 0.72). DNA extraction, SNP selection and genotyping was conducted as described in detail previously [33]. The success rate of genotyping was >95%. SNPs in the *GLP-1R gene* (Supplementary Table 2) were selected based on a recent study and were excluded if prior study report the minor allele frequency as <5% [68].

PLINK analysis [69] revealed that the SNPs did not differ from the Hardy–Weinberg Equilibrium (Supplementary Table 2).

#### Statistical analysis of the human genetic study

A linear mixed effect model in the MIXED procedure of SAS 9.3 (SAS Institute, Inc, Cary, NC, USA), was used. The analysis includes scores from all genotyped individual. This model adjusts for (i) the dependent nature of twin observations and (ii) dependence of individuals from the same family. Two separate variance–covariance matrices was modeled (monozygotic and dizygotic twins), as monozygotic and dizygotic twins share 100% and 50% of their genome respectively. Correlations between individuals in groups (1) and (2) were calculated with an R-side random effects with an unstructured variance–covariance matrix. The Bonferroni correction was used to control for multiple testing, where a primary analysis of 5 SNPs yielded a corrected alpha of 0.01.

## Results

### Acute Ex4 did not alter expression of aggressive behavior in male mice

In mice assigned to either vehicle or Ex4 treatment at the test day after aggression training (Fig. 1, Panel A) displayed a similar baseline attack latency during this period (Panel B; day 1–6). At test day, acute administration of Ex4 (2.4 µg/kg) did neither alter aggressive behaviors (Panel C, Fig. 1A–F) or non-aggressive (Panel D; Fig. 1G–J) behaviors compared to vehicle.

### Repeated treatment of Ex4 reduced the acquisition, but not expressing, of aggressive behaviors in male mice

As acute treatment did not alter aggressive behaviors, the effect of repeated Ex4 on aggressive behaviors was explored. Mice were treated during training (day 1–6), and on the test day (day 7) (Supplementary Fig. 1, Panel A). Compared to vehicle, repeated treatment of Ex4 (2.4 µg/kg) increased the latency to attack in these mice (Supplementary Fig. 1, Panel B). On the test day, there were no differences in aggressive behaviors (Supplementary Fig. 1, Panel C) or non-aggressive behaviors (Supplementary Fig. 1, Panel D) between Ex4 and vehicle treatment. These findings suggest that Ex4 reduced acquisition of aggression, but not the expression of aggression. Therefore, a different design was used onwards where the effect on repeated Ex4 injection on the acquisition of aggression was explored (Figs. 2 and 3, Panel A).

### Repeated treatment of Ex4 dose-dependently reduced the acquisition of aggressive behaviors in male mice

Subsequent experiments investigate the possibility that repeated Ex4 treatment dose-dependently (2.4 or 1.2 µg/kg) reduces the acquisition of aggressive behaviors.

Firstly, in mice with similar baseline aggression, repeated Ex4 (2.4 µg/kg) injection during training increased the latency to attack, a difference specifically evident at day 5 (Fig. 2A). This is further evident as the AUC for attack latency was higher in Ex4 treated mice compared to vehicle mice (Fig. 2B). At test day Ex4 did not alter attack duration (Fig. 2C), but reduced attack frequency (Fig. 2D) and increased attack latency (Fig. 2E). Despite a robust effect on attack behaviors, Ex4 did not alter threat duration, frequency or latency (Fig. 2F–H). When it comes to non-aggressive behaviors (Panel D), Ex4 did not affect social behavior duration (Fig. 2I), whereas it increased social behavior frequency (Fig. 2J), without altering the social behavior latency (Fig. 2K), or non-social behavior duration (Fig. 2L). In an attempt to define underlying mechanisms, the levels of monoaminergic neurotransmission in various aggression-related areas and the plasma levels of testosterone or corticosterone were investigated in these mice. Repeated Ex4 treatment did not alter the levels of monoamines, their metabolites or turnover in VTA, prefrontal cortex, hypothalamus, hippocampus or amygdala (Supplementary Table 3), but altered neurotransmission in NAc (Fig. 2, Panel E). Specifically, repeated Ex4 injections decreased noradrenalin, serotonin and 5-HIAA levels (Fig. 2M–O), without altering serotonin turnover, the levels of dopamine, DOPAC, 3-MT or dopamine turnover (Fig. 2P–T). Correlational analyses were conducted in an attempt to correlate Ex4-induced alterations in neurotransmission in NAc with the behavioral outcomes (Table 5). Noradrenalin levels correlated with attack latency and social behavior frequency. 5-HT levels correlated with attack latency and social behavior frequency, whereases 5-HIAA levels correlated with attack duration, attack frequency, attack latency, social behavior frequency and social behavior latency. Moreover, Ex4 did not alter the levels of corticosterone or (Fig. 2U, V) testosterone in plasma.

**Table 5:** Correlational analyses between levels of noradrenaline, serotonin or 5-HIAA in nucleus accumbens (NAc) and behaviors in male mice from the resident intruder test which were injected repeatedly with exendin-4 or vehicle.

	Noradrenalin in NAc (pmol/mg)		Serotonin in NAc (pmol/mg)		5-HIAA in NAc (pmol/mg)	
	Pearson *r* (95%, CI)	*P*-value	Pearson *r* (95%, CI)	*P*-value	Pearson *r* (95%, CI)	*P*-value
**Attack duration (s)**	0.2205 (−0.3512–0.6724)	0.4488	0.2932 (−0.2811–0.7129	0.3090	0.5675 (0.0528–0.8439)	0.0343*
**Attack frequency**	0.4209 (−0.1412–0.7778)	0.1340	0.3555 (−0.2157–0.7455)	0.2122	0.6113 (0.1195–0.8622)	0.0343*
**Attack latency (sec)**	−0.7434 (−0.9136–−0.3515)	0.0023**	−0.6237 (−0.8673–−0.1393)	0.0171*	−0.5461 (−0.8348–−0.0219)	0.0433*
**Threat duration(s)**	0.02718 (−0.5108–0.5498)	0.9265	0.1416 (−0.4206–0.6252)	0.6291	0.2559 (−0.3178–0.6925)	0.3772
**Threat frequency**	−0.0058 (−0.5348–0.5264)	0.9842	0.1243 (−0.435–0.6144)	0.6720	0.0222 (−0.5144–0.5463)	0.9400
**Threat latency (s)**	−0.3664 (−0.751–0.2038)	0.1975	−0.4223 (−0.7785–0.1395)	0.1325	−0.0299 (−0.5517–0.5087)	0.9191
**Social behavior duration (s)**	−0.2057 (−0.6638–0.3647)	0.4806	−0.2775 (−0.7043–0.2968)	0.3369	−0.1246 (−0.6145–0.4347)	0.6714
**Social behavior frequency**	−0.5559 (−0.839–−0.0359)	0.0390*	−0.5626 (−0.8419–−0.0457)	0.0362*	−0.7681 (−0.9227–−0.401)	0.0013**
**Social behavior latency (s)**	0.3023 (−0.2718–0.7178)	0.2934	0.256 (−0.3174–0.6927)	0.3763	0.5505 (0.0281–0.8367)	0.0414*
**Non-social behavior duration (s)**	−0.07643 (−0.5834–0.4733)	0.7951	−0.165 (−0.6396–0.4006)	0.5729	−0.5001 (−0.8145–0.0416)	0.0686

Data are presented with Pearson r together with the 95% confidence interval (CI) and the corresponding *P*-value for each correlational analysis. *P* < 0.05 is considered as statistically significant; *P < 0.05; **P < 0.01.

As demonstrated in Fig. 3 (Panel B), a low dose of Ex4 (1.2 µg/kg) during training did not alter the latency to attack (Fig. 3A) or the AUC (Fig. 3B). On the test day, a low dose of Ex4 decreased aggressive behaviors (Panel C). Ex4 did not alter attack duration, frequency or latency (Fig. 3C, D), but decreased threat duration and frequency (Fig. 3F, G) and increased threat latency (Fig. 3H). Besides, the scores of social behavior duration, social behavior frequency, social behavior latency or non-social behavior duration do not differ between treatment groups (Fig. 3I–L; Panel D).

Western blot studies (Fig. 3, Panel B; *n* = 3 per group) were conducted on these mice to further indicate that Ex4-reduced aggression involves serotoninergic and noradrenergic neurotransmission in NAc, specifically the core or shell compartment. Additionally, raphe was studied as it sends the main serotoninergic projection to NAc. These studies revealed that a low dose of Ex4 enhanced the TPH2 protein levels of raphe (*P* < 0.05, Fig. 3L–N). There were no observed differences in any investigated protein between treatments in NAc core (Fig. 3O, P). In NAc shell, the protein levels of DBH (*P* < 0.05), NET (*P* < 0.01), or SERT (*P* < 0.01) (Fig. 3Q, R) were lower in Ex4 compared to vehicle-treated mice. No differences were observed when it comes to GLP-1R or 5HT1B.

Immunohistochemical detection of neuronal activity in dorsal raphe (Panel F) reveal that the low dose of Ex4 did not alter the number of cells positive for FOS in this area (Fig. 3S, T, *P* = 0.1878). As Ex4 alters neurotransmission in NAc shell, we aimed to study the possibility that treatment alters neuronal activity by means of immunohistochemical studies (Fig. 3, Panel F). Similar to the western blot findings, the low dose of Ex4 did not alter the number of cells positive for FOS in NAc core (Fig. 3U, V, *P* = 7972). In contrast to the western blot studies, the low dose of Ex4 did not affect the number of FOS positive cells in NAc shell (Fig. 3U, W, *P* = 9289), indicating that treatment rather acts pre-synaptic or alters non-neuronal cells. As expected, there was not a difference in the number of cells positive for FOS immunoreactivity in PVT (Supplementary Fig. 2A, B), BNST (Supplementary Fig. 2C, D), or MPOA (Supplementary Fig. 2E, F). The real-time PCR data (Fig. 4, Panel F) reveal that the expression of *GLP-1R* in NAc (Fig. 4T) or dorsal raphe (Fig. 4U) was similar in mice exposed to HDF compared to those consuming chow.

### Exposure to HFD blunted the Ex4 reduced aggression in male

The final resident intruder test explores the possibility that As HFD impairs the ability of Ex4 to reduce aggressive behaviors (Fig. 4, Panel A).

The latency to attack latency (Fig. 4A) and AUC (Fig. 4B) were similar between mice feed peanut butter and treated repeatedly with Ex4 or vehicle (Panel B). As shown before for HFD [54], the 24-h intake of peanut butter did not differ between treatment groups (Fig. 4C), both during baseline (day 0–1) and during treatment (day 2–5). There were no differences in attack duration, attack frequency, attack latency, threat duration, threat frequency and threat latency between mice fed peanut butter and treated with either Ex4 or vehicle (Fig. 4D–I; Panel C). Additionally, Ex4 did not alter non-aggressive behaviors (Panel D; Fig. 4J–M).

To confirm that HFD blunts the responsiveness of Ex4 on aggression, the expression of serotonin and noradrenalin-related proteins were investigated in additional western blots studies (Fig. 4, Panel E) from these mice. In mice fed HFD, Ex4 enhanced the TPH2 protein levels of raphe (*P* < 0.05, Fig. 4N, O). As expected, there was no difference in the expression of any investigated protein in NAc core (Fig. 4P, Q) between the two treatment groups. In mice fed HFD, Ex4 lowered the expression of DBH (*P* < 0.05), and enhanced NET (*P* < 0.05), without altering any other investigated protein in NAc shell (Fig. 4R, S). Moreover, exposure to HFD does not change the expression of GLP-1R in NAc (Fig. 4T) or dorsal raphe (Fig. 4U).

### Human genetic data

In the human genetic study, none of the studied SNPs in the *GLP-1R gene* were associated with overt aggression (all *P* > 0.19).

## Discussion

Herein we report that repeated, but not acute, administration of Ex4 dose-dependently reduces the acquisition, but not expression, of aggression-like behaviors in male mice in the resident intruder test. As evident from neurochemical and western blot studies, this reduction correlates with neurotransmission of serotonin as well as noradrenalin in NAc, specifically the shell compartment. Besides, intake of an HFD blunts the ability of Ex4 to decrease aggression. Although this interaction is evident in male mice, no association between overt aggression in young men and polymorphisms in the *GLP-1R* genes was found.

As shown in various experiment herein, repeated Ex4 treatment during training increases the latency to attack, and thereafter suppresses attack behaviors and as a compensation increases social behaviors at test day. A similar effect is obtained by a lower dose of Ex4, where lower threat behaviors and tendency to increase attack latency is evident in Ex4 treated mice compared to those treated with vehicle. It may therefore be suggested that the anti-aggressive effects of Ex4 are more pronounced during acquisition rather than during full expression of the behavior. Supportively, an acute injection of Ex4 does not influence the expression of aggressive behaviors in mice already trained to be aggressive. This is further supported as the ability of Ex4 to reduce aggression disappears after a prolonged treatment schedule. The possibility that GLP-1 influences the acquisition but not expression should be determined in detail in upcoming studies. It should also be considered that tolerance to Ex4 may influence this outcome. Indeed, tolerance may be associated with a desensitization of the GLP-1R to Ex4, which already has been demonstrated in cell cultures [70]. The findings that Ex4 causes tolerance to other reward-related behaviors [71], an effect not evident by GLP1R agonists like dulaglutide [72], raises the need for additional aggression studies with dulaglutide.

As evident from the neurochemical and western blot studies, alterations in serotonergic and noradrenergic neurotransmission in NAc appear important for the ability of Ex4 to reduce aggression. Specifically, the NAc of chow-consuming mice with low aggression display lower ex vivo levels of noradrenaline, serotonin and 5-HIAA and in consistency lower protein levels of DBH, NET, and SERT after high and low dose of Ex4 treatment respectively. A behavioural-neurotransmission correlation is evident as both serotonin and 5-HIAA correlates to attack latency. However, the interpretation of these data is limited as a small number of animals were used in theses western blot studies. Given the preliminary outcome and putatively contrasting outcome of the present data, additional experiments deigned to define the exact circuits and mechanisms involved are thus warranted. The western blot findings imply that the shell compartment of NAc, rather than any other studied areas was associated with the Ex4 reduced aggression. Given that monoaminergic signaling in NAc is central for the reward of attack [15–18] raises the possibility that GLP-1R activation decreases aggressive behavior via a reduced reward of such behavior. In support for the contention that aggression involves reward are the findings that aggressive behaviors alters monoaminergic signaling within NAc [15–18, 73, 74] and an aggression provoking task activates NAc [75–78]. Supportively, a higher probability of future winning is associated with neuroplasticity in androgen and monoamine signaling in reward-processing areas in the brain [20, 79–82]. The possibility that Ex4 reduces the reward of aggression is further supported as systemic or brain infusion of Ex4 into reward-related areas decrease sexual behaviors [43, 44] and reward associated with addictive drugs and natural rewards (for review see [83]). Although intriguing, upcoming studies should investigate the influence of Ex4 in aggression paradigms more specifically reflecting reward such as aggression-induced condition place preference and operant aggression-seeking [1]. Although both neurochemical and western blot studies imply altered monoaminergic neurotransmission in NAc, no differences in the number of cells positive for FOS immunoreactivity in NAc shell between Ex4 and vehicle was reported. This may indicate that Ex4 treatment does not alter the neuronal activity in this area. As the aforementioned proteins are expressed on both neurons and astrocytes [26], upcoming tests should explore the possibility that astrocytes modulate interaction between Ex4, aggression, and serotonin/noradrenaline. Presynaptic effects are another tentative explanation that should be considered.

Although reward processing might influence the obtained data, other domains may influence the outcome of a complex behavior like aggression. One of these tentative mechanisms could be a suppressed locomotor activity which is unlikely as the selected Ex4 doses do not alter locomotor activity per se in the open field [39]. Another tentative explanation is that Ex4 alter explorative processes and subsequently aggression. That is unlikely since the resident-intruder test is performed in the home-cage of the resident mice where exploration is low. As all drugs were injected systemically, peripheral signals may participate in the ability of Ex4 to reduce aggressive behaviors. These may include testosterone and corticosterone, which previously have been associated with aggression [84–87]. However, this appears less likely as there were no treatment effect on these hormones. Another possibility is a reduced depressive state as repeated Ex4 decreases immobility in the forced-swim test [88, 89]. Anxiety is another confounding factor. Moreover, as the outcome of GLP-1R activation on anxiety-like behavior are inconsistent and depend on contextual factors [89–94] the possible influence on such behavior is complex. Although, upcoming studies should evaluate the complex association between anxiety state, aggression, contextual factors, and GLP-1 further.

In our pilot experiment with HFD exposure the aggression levels is similar between vehicle and Ex4-treated mice, indicating that continuous access to an HFD (peanut butter) blunts the ability of Ex4 to reduce aggression in male mice. On this note, HFD impairs the response to Ex4 when it comes to feeding or vagal afferent activation [53–55]. It should however be taken into consideration that the diet per se causes less aggression although this appears less likely as HFD rather enhances aggression in rodents [52]. Nonetheless, this cannot be ruled out as a non-HFD (chow) control condition was not included in this experiment. The possibility that vehicle-treated mice eat HFD rather than engage in fighting [59] is another tentative explanation to the blunted Ex4 repones after HFD. However, this is unlikely as neither vehicle or Ex4-treated mice eat HFD during any of the resident-intruder daily encounter. Neither did Ex4 treatment alter food intake outside the test, indicating that food-restriction does not influence the obtained data. The pilot nature of this study raises the concern that the interpretation of these experiments should be cautious and additional experiments should explore this interaction in detail. The western blot data further reveal that the protein levels of SERT in NAc shell returns to baseline in mice consuming HFD and treated with Ex4 compared to those consuming chow. This implies that serotonin in NAc shell may participate in the interaction between GLP-1, aggression and HFD. It should also be noted that the protein levels of TPH2 of raphe are elevated and DBH in NAc are lower in Ex4 mice in both chow and HFD consuming mice. Moreover, the NET levels are low versus high after Ex4 in mice after chow and HDF respectively. Therefore, these proteins are less likely involved in the HFD blunted Ex4 response on aggression, but present a diet-independent effect of GLP-1R stimulation. However, comparisons of the outcome between each of tests are limited by the use of two different Ex4 does, differences in aggression level and exposure to HFD in the latter, but not initial tests. Although the protein levels of GLP-1R is similar in both groups of mice, previous studies show that HFD alters the expression of GLP-1R mRNA on vagus [55]. It should thus be considered that HFD diet alters the expression of the GLP-1R, and thereby alters the response to Ex4. However, this appears less likely as HFD exposure does not alter the expression of the GLP-1R in NAc or dorsal raphe. On the contrary, HFD may alter the expression of GLP1-R on other brain regions or the expedition of GLP-1; a focus of upcoming studies. Another tentative explanation might be that Ex4 fails to reduce aggression as HFD reduces the reward thereof. Supportively, HFD exposure decreases rewards like palatable foods intake and drug reward [49–51]. Supportively, Ex4 does not alter the intake of foods considered rewarding (HFD) in the present study.

This study provides support for the role of Ex4 in the acquisition of aggression behaviors, however several limitations should be taken into consideration. First, it should be considered a limitation that Ex4, although known to pass the blood-brain-barrier [95, 96], always was injected systemically. Moreover, inter-batch and intra-batch variation in behaviors exist which might influence the obtained results. To minimize this possible confounder, vehicle controls are included and treatment were either randomized (experiment 2) or stratified based on acquired aggression level (experiment 1) and innate aggression level (experiment 3,4,5). Inter-batch variation may also influence the outcome of the data. The low innate aggression levels in the C57Bl6/N strain of mice, thus including mice with no aggression on day 1, may confound the data but appropriate stratification and vehicle controls are included to minimize this confounder. Therefore, strict comparison between experiments is difficult. Herein, we show that a low, but not high dose of Ex4 attenuates threat behavior. These differences in behaviors may be linked to dose-dependent effects of Ex4 treatment, but may also be attributed to inter-batch variation in aggression levels. The current experiments was performed in male mice and as aggressive behavior is an innate sexually dimorphic behavior most commonly studied in males (for review see ref. [97]). However, the lack of inclusion of females should be considered as a major limitation with the present study. Notably, in contrast to C57Bl6 female mice lactating or gestational females, female hamsters, female California or Swiss webster mice or female rats display aggression in the female intruder test [9, 10, 98–101]. Hence, upcoming studies should investigate the role of Ex4 on aggression in such female species and strains as this would further contribute to the understanding of the neurobiological underpinnings of female aggression compared to intermale aggression.

Here, we found no associations between polymorphisms in the *GLP-1R* genes and overt aggression in young men of the CATSS cohort, indicating that genetic alterations of GLP-1R does not influence the expression of aggression. However, several limitations such as small sample size and that only young males with Caucasian descent were included may have contributed to the negative outcome. Contrarily, a link between a studied *GLP1R* gene polymorphisms is associated with AUD, increased alcohol intravenous intake and higher BOLD response in the right globus pallidus in response to notification of high monetary reward [68]. Thus, we conclude that genetic variation of GLP-1R genes does not contribute to vulnerability to engage in overt aggression, which is supported by the preclinical data where Ex4 does not alter the expression in mice with an established behavior. However, as we further report that Ex4 attenuates the acquisition of aggression in male mice, the role of these *GLP-1R* polymorphisms in the acquisition of aggressive behaviors should be studied in men.

The pharmacological studies of male mice show that GLP-1R activation inhibits the acquisition of aggressive-like behaviors. It might be suggested that these findings may have a clinical application as the GLP-1R agonist, liraglutide, decreases aggressive behaviors in a man with autism-spectrum disorder [102]. On the other hand, GLP-1R may be a less suitable target to treat aggression in humans as GLP-1R activation did not alter expression of aggression in male mice with an established behavior. On this note, the results from the genetic study indicate that GLP-1R may have limited importance for the regulation of the expression of aggression in men. Despite this, additional studies are warranted to gain more mechanistic insight into the anti-aggressive properties of GLP-1R activation.

## Supplementary information

highlights

supplementary Table 1–3

supplementary Fig. 1–2
