# Supplementary material for: Activation of glucagon-like peptide-1 receptors reduces the acquisition of aggression-like behaviors in male mice
Source: Transl Psychiatry. 2022 Oct 13;12:445. doi: 10.1038/s41398-022-02209-0 (PMC9561171; doi:10.1038/s41398-022-02209-0)
Supplement: Supplementary file 1 — highlights [file 41398_2022_2209_MOESM1_ESM.docx]

# Highlights

- Repeated, but not acute, Ex4 treatment dose-dependently reduced aggressive behaviors.

- This involves serotonergic and noradrenergic signaling in nucleus accumbens.

- High-fat diet blunts the ability of Ex4 to reduce aggressive behaviors

- No association between polymorphisms of the *GLP-1*-related genes and overt aggression.

- GLP-1 signaling suppresses aggressive behaviors via central neurotransmission.
