# Supplementary material for: Activation of glucagon-like peptide-1 receptors reduces the acquisition of aggression-like behaviors in male mice
Source: Transl Psychiatry. 2022 Oct 13;12:445. doi: 10.1038/s41398-022-02209-0 (PMC9561171; doi:10.1038/s41398-022-02209-0)
Supplement: Supplementary file 2 — supplementary Table 1–3 [file 41398_2022_2209_MOESM2_ESM.docx]

**Supplementary table 1. Items of the self-Reported Delinquency questionnaire for overt aggression**

| **Overt aggression Item** | **Content of item** |
| --- | --- |
| **1** | Threatened, hassled or bullied someone |
| **2** | Started a fight |
| **3** | Used violence or threatened someone to get money or an article of value |
| **4** | Beaten or hurt an animal |
| **5** | Beaten or hurt a person |
| **6** | Beaten or hurt someone so that he/she needed medical care |
| **7** | Hurt someone with a baseball bat, a knife or another weapon |
| **8** | Had sex with someone against their will by making use of your influence or giving the person alcohol or drugs |
| **9** | Had sex with someone against their will by threatening or using physical coercion or violence |

All included participants reported the frequency over the past 12 months of each item; 0 (never), 1 (1-2 times), 2 (3-5 times), 3 (6-10 times), or 4 (more than 10 times).

**Supplementary table 2. Description of the genotyped SNPs of the CATSS cohort.**

| **SNP** | **Gene** | **SNP location** | **SNP type** | **minor allele** | **major allele** | **genotype counts** | **MAF** | **HWE p-value** |
| --- | --- | --- | --- | --- | --- | --- | --- | --- |
| **rs7766663** | ***GLP1R*** | **chr6:39068006** | **Intron** | **T** | **G** | **220/373/164** | **0.46** | **0.97** |
| **rs7341356** | ***GLP1R*** | **chr6:39068569** | **Intron** | **G** | **A** | **213/376/177** | **0.48** | **0.91** |
| **rs2235868** | ***GLP1R*** | **chr6:39072878** | **Missense** | **G** | **T** | **198/374/185** | **0.49** | **0.95** |
| **rs7769547** | ***GLP1R*** | **chr6:39080981** | **Intron** | **A** | **G** | **240/370/147** | **0.44** | **0.98** |
| **rs10305512** | ***GLP1R*** | **chr6:39086337** | **Non coding transcript variant** | **A** | **G** | **704/67** | **0.04** | **0.45** |

SNP = Single Nucleotide Polymorphism; *GLP1R* = glucagon-like peptide-1 gene; MAF = minor allele frequency; HWE = Hardy-Weinberg equilibrium. Genotype counts are represented as the common homozygote/heterozygote/uncommon homozygote.

**Supplementary table 3. *Ex vivo* data of monoamines, metabolites and turnover in selected brain areas**

|  | **VTA** | | | **PFC** | | | **Hippocampus** | | | **Amygdala** | | | **Hypothalamus** | | |
| --- | --- | --- | --- | --- | --- | --- | --- | --- | --- | --- | --- | --- | --- | --- | --- |
|  | **Ex4**  **n=6** | **Veh**  **N=7** | **Unpaired**  **t-test** | **Ex4**  **n=7** | **Veh**  **N=7** | **Unpaired**  **t-test** | **Ex4**  **n=7** | **Veh**  **N=7** | **Unpaired**  **t-test** | **Ex4**  **n=7** | **Veh**  **N=7** | **Unpaired**  **t-test** | **Ex4**  **n=6** | **Veh**  **N=7** | **Unpaired**  **t-test** |
| **5-HT**  **(pmol/mg)** | 1.5 ±  0.2 | 1.2 ±  0.1 | t(11)=1.29,  P=0.2225 | 1.9 ± 0.2 | 2.2 ±  0.3 | t(12)=0.68,  P=0.5076 | 2.6 ±  0.3 | 2.6 ±  0.1 | t(12)=0.23,  P=0.8247 | 2.9 ±  0.8 | 3.1 ±  0.6 | t(12)=0.25,  P=0.8067 | 3.9 ±  0.4 | 3.6 ±  0.3 | t(11)=0.60,  P=0.5586 |
| **5-HIAA**  **(pmol/mg)** | 0.8 ±  0.1 | 0.6 ±  0.1 | t(11)=1.08,  P=0.3054 | 0.7 ± 0.1 | 0.7 ±  0.1 | t(12)=0.61,  P=0.5515 | 1.1 ±  0.1 | 1.0 ±  0.1 | t(12)=0.17,  P=0.8687 | 1.4 ±  0.2 | 1.7 ±  0.2 | t(12)=0.95,  P=0.3631 | 1.5 ±  0.2 | 1.6 ±  0.1 | t(11)=0.25,  P=0.8095 |
| **5-HIAA/**  **5-HT** | 0.5 ±  0.0 | 0.6 ±  0.1 | t(11)=0.49,  P=0.6367 | 0.4 ± 0.0 | 0.4 ±  0.0 | t(12)=0.15,  P=0.8859 | 0.4 ±  0.0 | 0.4 ±  0.0 | t(12)=0.31,  P=0.7588 | 0.7 ±  0.1 | 0.6 ±  0.1 | t(12)=0.16,  P=0.8791 | 0.4 ±  0.1 | 0.4 ±  0.0 | t(11)=0.58,  P=0.5756 |
| **Noradrenalin**  **(pmol/mg)** | 0.1 ±  0.3 | 0.6 ±  0.1 | t(11)=1.29,  P=0.2253 | 1.7 ± 0.3 | 1.8 ±  0.3 | t(12)=0.37,  P=0.7145 | 2.5 ±  0.2 | 2.8 ±  0.2 | t(12)=1.07,  P=0.3077 | 2.9 ±  0.9 | 4.0 ±  1.2 | t(12)=0.73,  P=0.4817 | 9.8 ±  0.7 | 8.0 ±  0.7 | t(11)=1.94,  P=0.0786 |
| **Dopamine**  **(pmol/mg)** | 0.1 ±  0.05 | 0.06 ±  0.02 | t(11)=1.64,  P=0.1304 | 0.3 ± 0.1 | 0.3 ±  0.1 | t(12)=0.52,  P=0.6151 | 0.07 ±  0.02 | 0.07 ± 0.01 | t(12)=0.20,  P=0.8484 | 0.4 ±  0.2 | 0.6 ±  0.4 | t(12)=0.54,  P=0.5978 | 1.3 ±  0.2 | 1.2 ±  0.1 | t(11)=0.33,  P=0.7500 |
| **DOPAC**  **(pmol/mg)** | n.d. | n.d. |  | 0.1 ± 0.0 | 0.07 ±  0.02 | t(12)=1.55,  P=0.1475 | n.d. | n.d. |  | 0.2 ±  0.1 | 0.2 ±  0.1 | t(12)=0.31,  P=0.7655 | 0.2 ±  0.1 | 0.1 ±  0.0 | t(11)=0.45,  P=0.6612 |
| **DOPAC/**  **Dopamine** | n.d. | n.d. |  | 0.4 ± 0.0 | 0.4 ±  0.1 | t(12)=0.13,  P=0.9025 | n.d. | n.d. |  | 1.2 ±  0.5 | 0.7 ±  0.2 | t(12)=0.93,  P=0.3725 | 0.2 ±  0.1 | 0.1 ±  0.0 | t(11)=0.64,  P=0.5354 |

In mice exposed to the resident-intruder paradigm, repeated exendin-4 (Ex4) does not alter monoaminergic neurotransmission in the ventral tegmental area (VTA), prefrontal cortex (PFC), hippocampus, amygdala or hypothalamus compared to vehicle (Veh) treatment. Serotonin (5-HT); 5-hydroxyindoleacetic acid (5-HIAA); 3,4-dihydroxyphenylacetic acid (DOPAC); Not detected=n.d.
