# Supplementary material for: Activation of glucagon-like peptide-1 receptors reduces the acquisition of aggression-like behaviors in male mice
Source: Transl Psychiatry. 2022 Oct 13;12:445. doi: 10.1038/s41398-022-02209-0 (PMC9561171; doi:10.1038/s41398-022-02209-0)
Supplement: Supplementary file 3 — supplementary Fig. 1–2 [file 41398_2022_2209_MOESM3_ESM.pptx]

## Slide 1
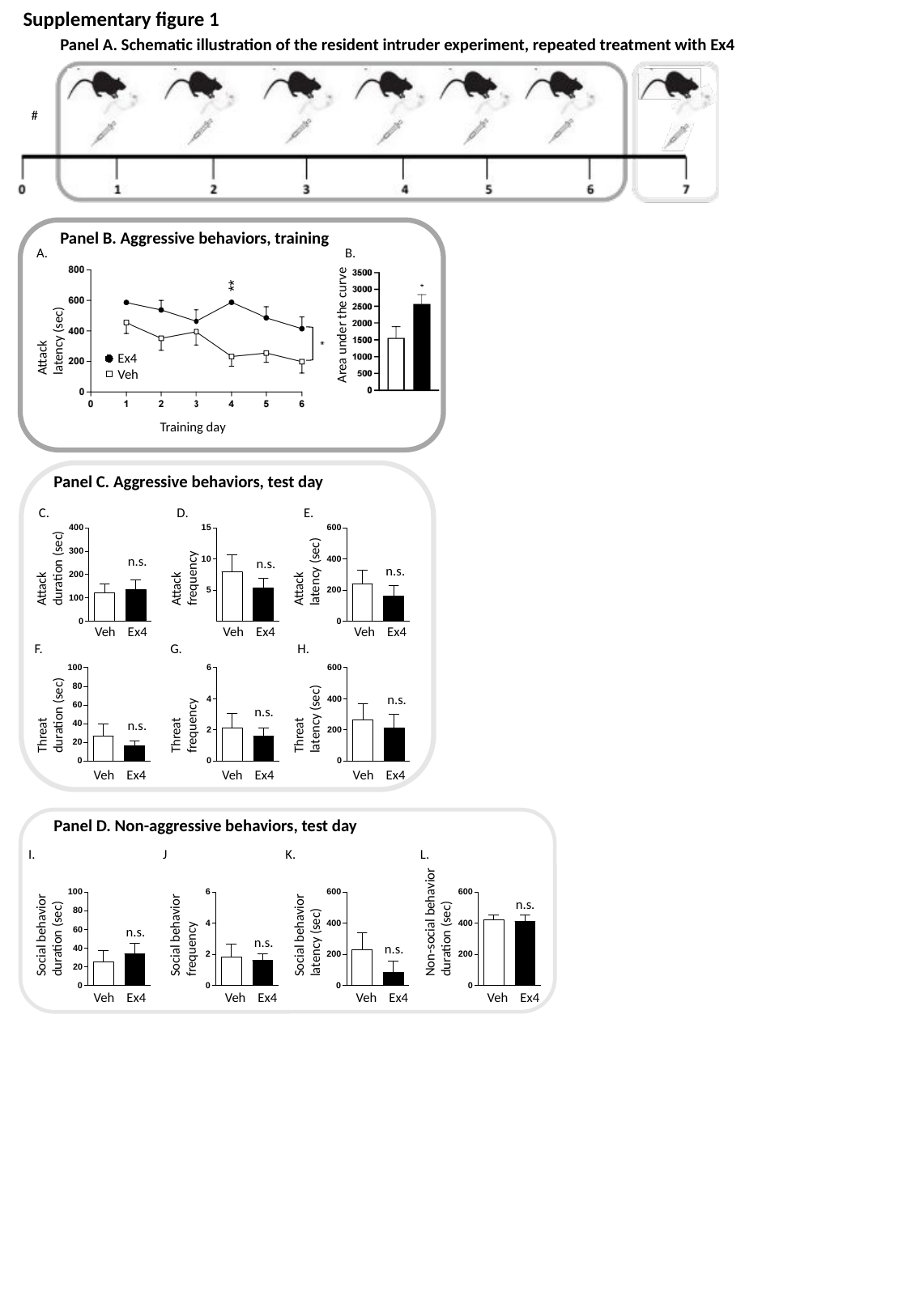

Supplementary figure 1
 Panel A. Schematic illustration of the resident intruder experiment, repeated treatment with Ex4
 Panel B. Aggressive behaviors, training
 Panel C. Aggressive behaviors, test day
 Panel D. Non-aggressive behaviors, test day
#
A. B.
**
Attack
latency (sec)
Area under the curve
*
Ex4
Veh
 Training day
C. D. E.
Attack
duration (sec)
Attack
frequency
Attack
latency (sec)
n.s.
n.s.
n.s.
 Veh Ex4 Veh Ex4 Veh Ex4
F. G. H.
Threat
duration (sec)
Threat
latency (sec)
Threat
frequency
n.s.
n.s.
n.s.
 Veh Ex4 Veh Ex4 Veh Ex4
I. J K. L.
Non-social behavior
duration (sec)
n.s.
Social behavior
duration (sec)
Social behavior
frequency
Social behavior
latency (sec)
n.s.
n.s.
n.s.
 Veh Ex4 Veh Ex4 Veh Ex4 Veh Ex4

## Slide 2
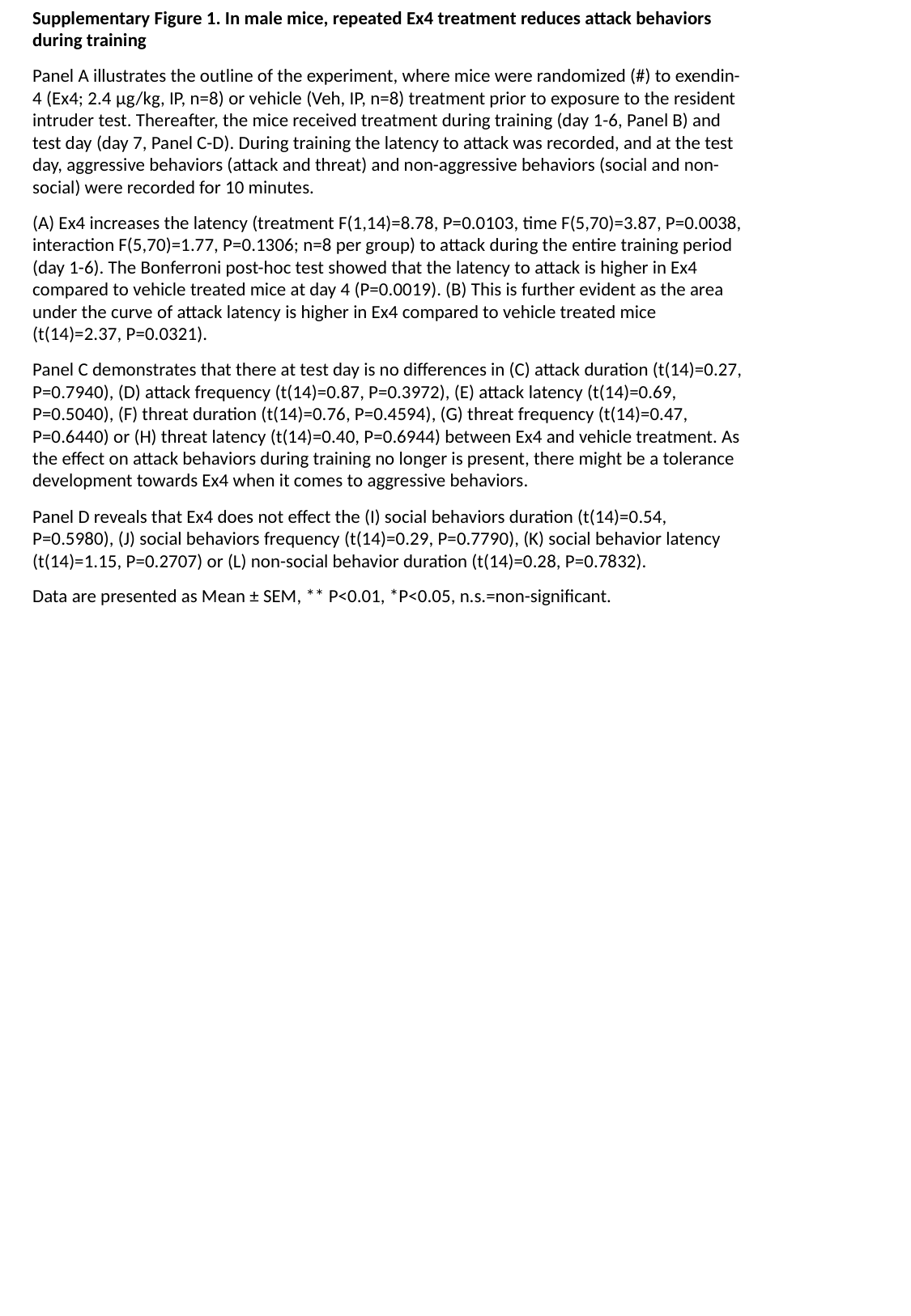

Supplementary Figure 1. In male mice, repeated Ex4 treatment reduces attack behaviors during training
Panel A illustrates the outline of the experiment, where mice were randomized (#) to exendin-4 (Ex4; 2.4 µg/kg, IP, n=8) or vehicle (Veh, IP, n=8) treatment prior to exposure to the resident intruder test. Thereafter, the mice received treatment during training (day 1-6, Panel B) and test day (day 7, Panel C-D). During training the latency to attack was recorded, and at the test day, aggressive behaviors (attack and threat) and non-aggressive behaviors (social and non-social) were recorded for 10 minutes.
(A) Ex4 increases the latency (treatment F(1,14)=8.78, P=0.0103, time F(5,70)=3.87, P=0.0038, interaction F(5,70)=1.77, P=0.1306; n=8 per group) to attack during the entire training period (day 1-6). The Bonferroni post-hoc test showed that the latency to attack is higher in Ex4 compared to vehicle treated mice at day 4 (P=0.0019). (B) This is further evident as the area under the curve of attack latency is higher in Ex4 compared to vehicle treated mice (t(14)=2.37, P=0.0321).
Panel C demonstrates that there at test day is no differences in (C) attack duration (t(14)=0.27, P=0.7940), (D) attack frequency (t(14)=0.87, P=0.3972), (E) attack latency (t(14)=0.69, P=0.5040), (F) threat duration (t(14)=0.76, P=0.4594), (G) threat frequency (t(14)=0.47, P=0.6440) or (H) threat latency (t(14)=0.40, P=0.6944) between Ex4 and vehicle treatment. As the effect on attack behaviors during training no longer is present, there might be a tolerance development towards Ex4 when it comes to aggressive behaviors.
Panel D reveals that Ex4 does not effect the (I) social behaviors duration (t(14)=0.54, P=0.5980), (J) social behaviors frequency (t(14)=0.29, P=0.7790), (K) social behavior latency (t(14)=1.15, P=0.2707) or (L) non-social behavior duration (t(14)=0.28, P=0.7832).
Data are presented as Mean ± SEM, ** P<0.01, *P<0.05, n.s.=non-significant.

## Slide 3
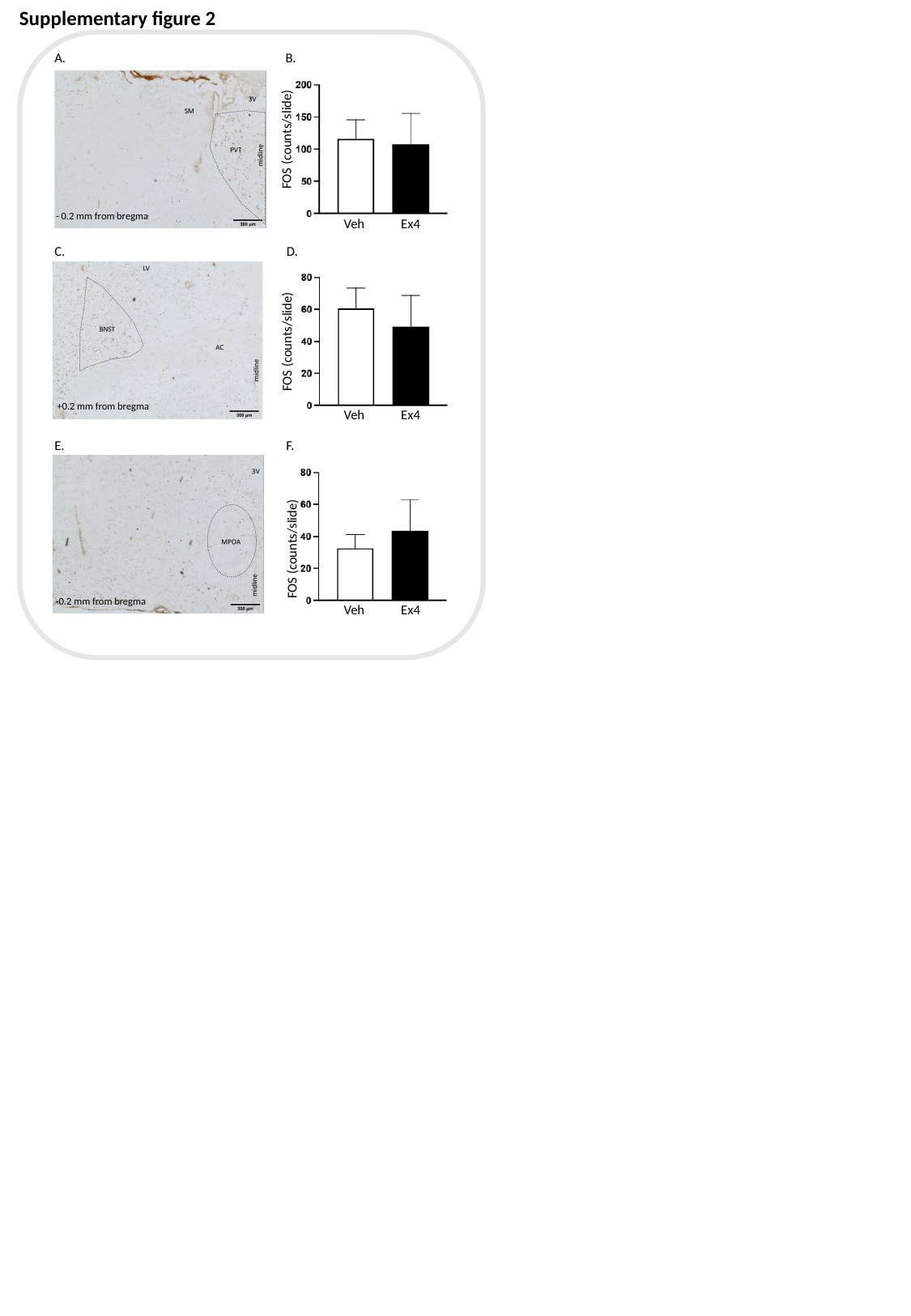

Supplementary figure 2
 B.
C. D.
E. F.
FOS (counts/slide)
- 0.2 mm from bregma
 Veh Ex4
FOS (counts/slide)
+0.2 mm from bregma
 Veh Ex4
FOS (counts/slide)
-0.2 mm from bregma
 Veh Ex4

## Slide 4
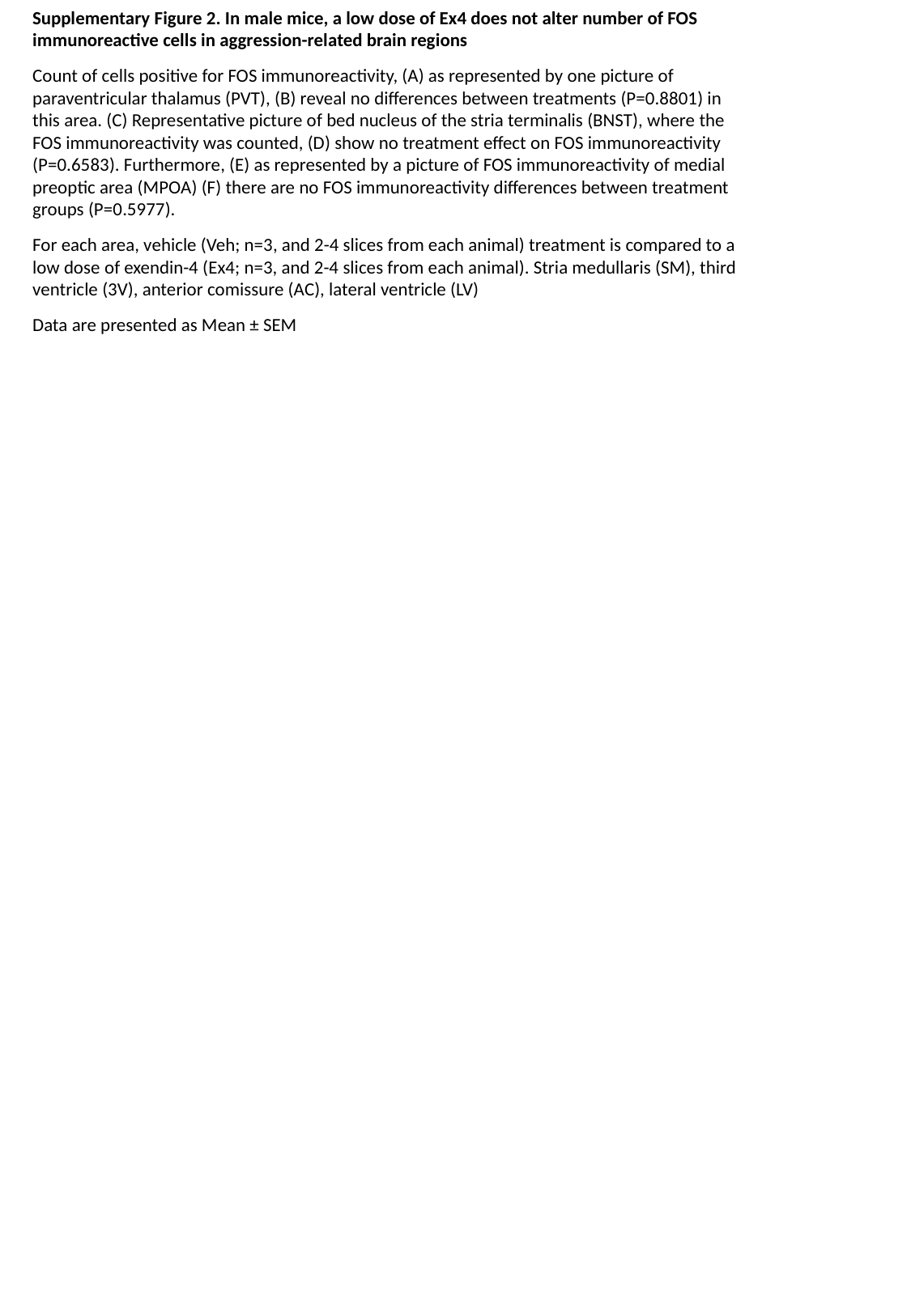

Supplementary Figure 2. In male mice, a low dose of Ex4 does not alter number of FOS immunoreactive cells in aggression-related brain regions
Count of cells positive for FOS immunoreactivity, (A) as represented by one picture of paraventricular thalamus (PVT), (B) reveal no differences between treatments (P=0.8801) in this area. (C) Representative picture of bed nucleus of the stria terminalis (BNST), where the FOS immunoreactivity was counted, (D) show no treatment effect on FOS immunoreactivity (P=0.6583). Furthermore, (E) as represented by a picture of FOS immunoreactivity of medial preoptic area (MPOA) (F) there are no FOS immunoreactivity differences between treatment groups (P=0.5977).
For each area, vehicle (Veh; n=3, and 2-4 slices from each animal) treatment is compared to a low dose of exendin-4 (Ex4; n=3, and 2-4 slices from each animal). Stria medullaris (SM), third ventricle (3V), anterior comissure (AC), lateral ventricle (LV)
Data are presented as Mean ± SEM
